# Higher pre-treatment lymphocyte-to-monocyte ratio predicts better tumor shrinkage after external beam radiotherapy in locally advanced cervical cancer

**DOI:** 10.3389/fonc.2026.1917302

**Published:** 2026-07-22

**Authors:** Lu Qiu, Yimin Li, Youjia Wang, Yuanfu Xie, Yanhong Zhuo

**Affiliations:** Department of Radiation Oncology, Zhangzhou Affiliated Hospital of Fujian Medical University, Zhangzhou, China

**Keywords:** cervical cancer, external beam radiotherapy, inflammatory marker, lymphocyte-to- monocyte ratio, radiotherapy response, retrospective cohort study, tumor shrinkage

## Abstract

**Background:**

The tumor shrinkage rate in patients with locally advanced cervical cancer after external beam radiotherapy varies widely, and simple pretreatment predictors are lacking. The lymphocyte-to-monocyte ratio (LMR) is a routine blood marker reflecting inflammatory and immune status; however, its association with short-term radiotherapy response in cervical cancer remains unclear.

**Methods:**

We retrospectively enrolled 247 patients with locally advanced cervical cancer who underwent radical external-beam radiotherapy between April 2020 and April 2025. LMR was calculated from pretreatment blood counts, with extreme values winsorized. The primary outcome was the continuous tumor shrinkage rate and the secondary outcome was a marked response (≥70% shrinkage). Multivariable linear and logistic regressions were used to assess the LMR-shrinkage association, adjusting for age, body mass index, International Federation of Gynecology and Obstetrics stage, pretreatment maximum tumor diameter, lymph node metastasis, concurrent chemoradiotherapy, and radiotherapy dose. Subgroup and sensitivity analyses were also performed.

**Results:**

In the multivariate analysis, each unit increase in LMR was associated with a 0.018 absolute increase in shrinkage rate (95% confidence interval (CI)], 0.001–0.036; P = 0.0477). The odds ratio for achieving a marked response was 1.117 per unit increase in LMR (95% CI: 1.002–1.356, P = 0.0241). The LMR effect was consistent across subgroups and remained significant after adjusting for nutritional and oxidative stress markers. The restricted cubic splines exhibit linearity (P for nonlinearity = 0.098).

**Conclusion:**

A higher pretreatment LMR was independently associated with better tumor shrinkage after external beam radiotherapy in patients with locally advanced cervical cancer. This readily available hematological marker may help identify patients who are likely to benefit from radiotherapy, although prospective validation is warranted.

## 1 Introduction

The global burden of cervical cancer is also substantial. Cervical cancer is the fourth most common cancer among women, with approximately 600,000 incident cases and 340,000 deaths annually. More than 85% of this burden affects low- and middle-income countries (1). The incidence of cervical cancer in China exhibits an upward trend and a shift toward younger demographics (2). For locally advanced patients, concurrent chemoradiotherapy is the standard treatment, in which external pelvic irradiation plays a crucial role in the overall efficacy. However, not all patients are clinically sensitive to radiotherapy, resulting in a significant incidence of treatment failure and recurrence (3). Identifying poor radiotherapy responders in advance and administering more targeted treatments is a practical clinical problem.

At present, the evaluation of radiotherapy response mainly relies on morphological comparison of MRI or CT before and after treatment. This method can only reflect the outcomes after the end of treatment and cannot stratify patients before treatment. The ICRU Report No. 89 proposed the concept of “adaptive radiotherapy,” in which the subsequent treatment plan is adjusted according to tumor regression after external beam irradiation, emphasizing that the tumor shrinkage rate is an important early indicator of radiosensitivity (4). Based on this concept, a number of clinical studies have used a high threshold of tumor shrinkage rate (mostly between 70% and 90%) to define a “radiotherapy- significant response” and have confirmed that patients who meet this criterion have a better long-term prognosis. Sun et al. established tumor volume reduction rate as an independent and powerful parameter for patients undergoing concurrent chemoradiotherapy, facilitating the identification of high-risk patients and guiding treatment adjustments (5). Choi et al. confirmed that a reduction of ≥81.8% was associated with significantly longer 3-year progression-free survival (75.4% vs. 36.2%, P < 0.001) and overall survival (93.2% vs. 69.0%, P = 0.002) (6). More recently, Lin et al. extended these findings by demonstrating that a reduction of ≥94% correlated with significantly improved 5-year outcomes (7). This means that if we can predict which patients may have an unsatisfactory tumor shrinkage rate before external irradiation, we can intervene in advance with intensive treatments (such as immunotherapy and targeted therapy). Our team previously found that nimotuzumab combined with chemoradiotherapy can improve the tumor shrinkage rate in elderly patients (8); however, it is not suitable for general use because of its high price and toxicity. Therefore, it is necessary to identify simple and low-cost predictors.

Inflammatory immune indicators in the peripheral blood routine meet these requirements, and their value in predicting treatment outcomes has been increasingly recognized in cervical cancer (9). For example, the lymphocyte-to-monocyte ratio (LMR) can be computed conveniently using standard hematological tests (10). Lymphocytes are key effector cells that orchestrate anti-tumor immunity (11). Radiotherapy not only directly kills tumors but also activates the immune response of the body (12). Upon entering the tumor, monocytes give rise to tumor-associated macrophages that generally mediate immunosuppressive effects and promote radioresistance (13, 14). Therefore, decreased LMR (lymphopenia or monocytosis) may indicate weakened anti-tumor immunity and enhanced immunosuppression, which may in turn affect the efficacy of radiotherapy. This view has been supported by evidence in other cancers such as nasopharyngeal carcinoma and esophageal cancer, which has shown that the level of LMR before treatment is related to the prognosis of radiotherapy and chemotherapy (15, 16). In the field of cervical cancer, most existing studies have focused on the relationship between LMR and long-term survival (17, 18), while analysis of early tumor regression after external beam radiotherapy is still rare.

To this end, the present study retrospectively collected data from patients with cervical cancer who underwent radical external beam radiotherapy at our institution and analyzed the association between pretreatment LMR levels and post-external beam radiotherapy (EBRT) tumor shrinkage rates. We anticipate that these findings may offer a readily available and inexpensive reference index for clinical assessment of radiosensitivity.

## 2 Materials and methods

### 2.1 Study population and data sources

**Figure 1:**
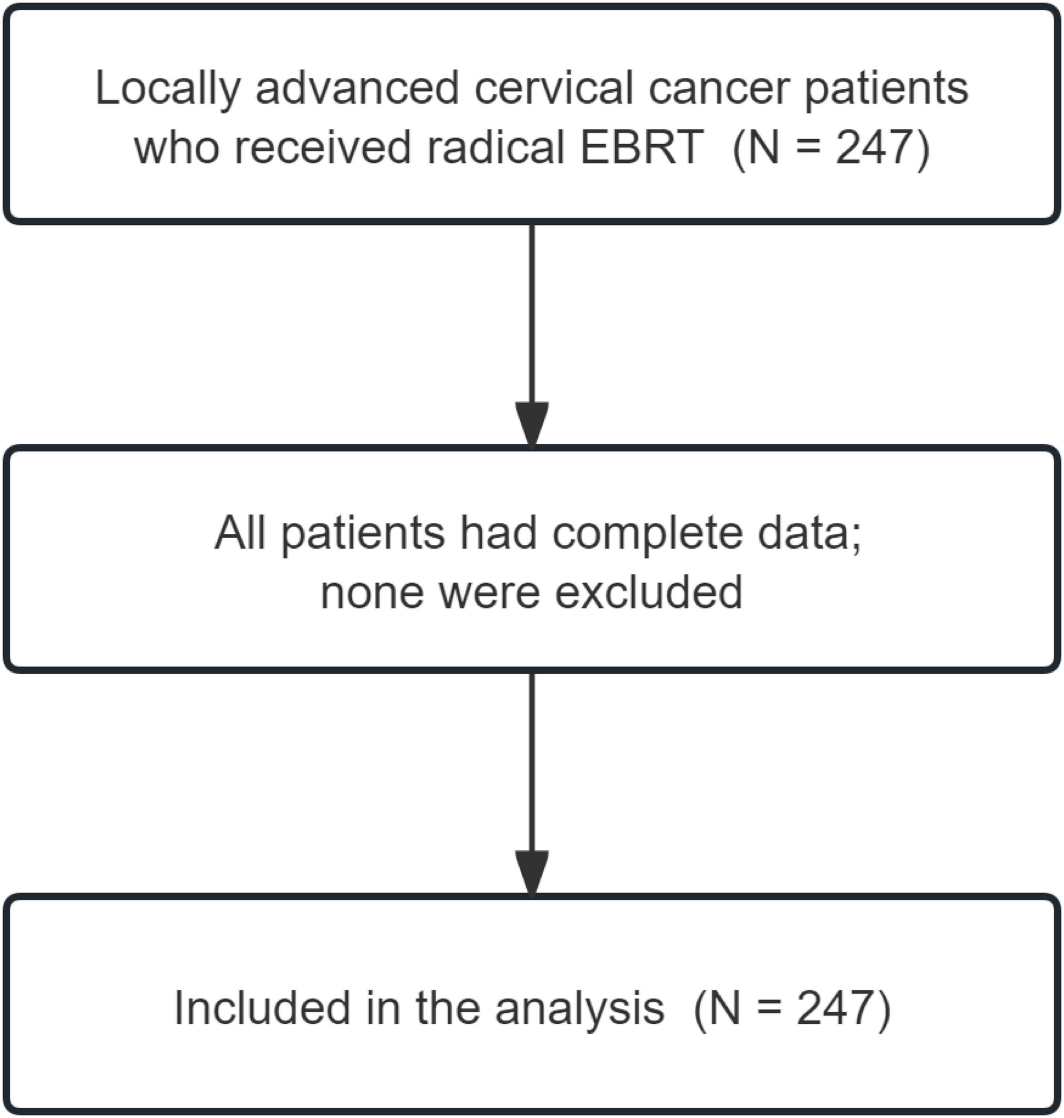
Flowchart of patient screening and selection. A total of 247 patients with locally advanced cervical cancer who underwent radical external beam radiotherapy (EBRT) and had complete clinical, imaging, and laboratory data met the inclusion criteria and were included in the final analysis.

Patients with International Federation of Gynecology and Obstetrics (FIGO) 2018 stage IIA–IVA (locally advanced) cervical cancer who underwent radical EBRT in our department between April 2020 and April 2025 were enrolled in this study. The included patients were required to meet the following criteria: (1) complete radical radiotherapy; (2) underwent pelvic enhanced MRI before and after EBRT, with good image quality; (3) had complete clinical, imaging, and laboratory data; and all planned analysis variables had no missing values. The following patients were excluded: (1) those with distant metastasis; (2) those who had previously undergone cervical surgery or radiotherapy; and (3) those with other severe comorbidities (such as severe heart and lung diseases). After applying these criteria, 247 patients with complete data were enrolled in this study. The screening process is illustrated in **Figure 1**.

### 2.2 Ethics statement

Given the retrospective design and use of de-identified data, informed consent was not required for this study, which was approved by the Ethics Committee of the Zhangzhou Affiliated Hospital of Fujian Medical University (Approval No. 2026LWB218).

### 2.3 Data collection and variable definitions

Data on each patient were retrieved from the electronic medical record system, including demographics and clinical characteristics such as age, body mass index (BMI), and FIGO stage (2018 version), and the presence or absence of lymph node metastasis; Treatment-related variables included whether to receive concurrent chemoradiotherapy and external beam radiation dose. Regarding the radiotherapy technique, 226 patients (91.5%) received intensity-modulated radiotherapy (IMRT) and 21 patients (8.5%) received three-dimensional conformal radiotherapy (3D-CRT). Peripheral blood samples were collected in the morning after overnight fasting, typically within 1–3 days before treatment initiation. Laboratory parameters included routine blood and liver function tests (gamma-glutamyl transferase and albumin levels).

For the assessment of tumor burden, the maximum tumor diameter before and after treatment was extracted from the pelvic contrast-enhanced MRI reports, all of which followed the RECIST 1.1 standard. The extracted data were checked and confirmed by two senior radiation oncologists in our department. To verify the accuracy of data entry, we randomly selected 30 cases and compared the original reports with the entered data individually, and no entry errors were found. In addition, all imaging reports in our hospital were first and final reviewed by radiologists, which further ensured the accuracy of the measurement data. Post-treatment MRI was performed within one week after the completion of EBRT and before brachytherapy to evaluate tumor shrinkage.

The composite index was calculated as follows: LMR was equal to the absolute value of lymphocytes divided by the absolute value of monocytes (×10^9^/L); The prognostic nutritional index (PNI) was computed using the following formula: serum albumin (g/L) + 5 × absolute lymphocyte count (×10^9^/L).

The primary endpoint was the rate of tumor regression after EBRT, which was calculated as a continuous variable ranging from 0 to 1 using the maximal-diameter method of RECIST 1.1 (maximum diameter before treatment minus maximum diameter after treatment) divided by the maximum diameter before treatment.

### 2.4 Statistical analysis methods

Data imputation was not performed because there was no missing data for any of the variables. Winsorization of the 1st and 99th percentiles was applied to the LMR to mitigate the impact of extreme outliers. Specifically, values below the 1st percentile (1.05) were set to 1.05, and values above the 99th percentile (9.69) were set to 9.69. Winsorized LMR was used for all subsequent analyses. After winsorization, a boxplot (**Supplementary Figure 1**) revealed no outliers beyond 1.5 times the interquartile range, indicating effective handling of extreme values. Continuous variables were tested for normality. Normally distributed variables were summarized as mean ± SD and compared using the t-test. Non-normally distributed data were reported as median interquartile range (IQR), with group differences evaluated using the Mann–Whitney U test. Categorical data are presented as absolute and relative frequencies and were analyzed using the χ² test.

We employed multivariable linear regression with stepwise adjustment to evaluate the independent relationship between LMR and continuous tumor shrinkage rate. Although the tumor shrinkage rate is theoretically bounded between 0 and 1, the observed values in our cohort were broadly distributed (median 0.6, IQR 0.4–0.8), with no substantial floor or ceiling effects. Residual diagnostic plots did not reveal any major violation of normality or homoscedasticity. Therefore, we considered linear regression appropriate for the primary analysis. To further verify the robustness of our results, we performed a sensitivity analysis using a logit-transformed outcome [log(TSR/(1-TSR))], where TSR denotes the tumor shrinkage rate, with the same covariate adjustment as in Model 3. A small offset (0.001) was added to both the numerator and denominator for cases with shrinkage rates at the boundaries (0 or 1) to avoid undefined values during logit transformation. The findings are presented as β-coefficients and 95% confidence intervals (CI). Four regression models with progressively adjusted covariates were built: crude (unadjusted); adjusted for age and BMI; additionally adjusted for stage, maximum pre-treatment tumor diameter, and lymph node metastasis; and further adjusted for concurrent chemotherapy status and radiation dose. A tumor shrinkage rate ≥ 70% was defined as a significant response to radiotherapy (good response). This threshold refers to the range commonly used in previous studies to define a significant response to radiotherapy (6, 7, 19)). Multivariate logistic regression with the same covariates as in Model 3 was used to assess the association between LMR and significant radiotherapy response. ROC analysis was also performed to determine how well the LMR could separate patients with marked shrinkage from those without, with the optimal cutoff determined using the Youden index.

To verify the linear relationship between the LMR and tumor shrinkage rate, restricted cubic spline (RCS) regression was performed with three knots located at the 5th, 50th, and 95th percentiles of the LMR distribution, adjusting for all covariates in Model 3. The P-value for nonlinearity was calculated.

We prespecified three subgroups: age (<65 vs. ≥65 years, separated by the median), FIGO stage (IIA-IIB vs. IIIA-IVA), and concurrent chemotherapy (no vs. yes). Within each subgroup, we applied the same regression model as in Model 3 to estimate LMR’s β and 95% CI of the LMR. To test for interactions, we introduced product terms (LMR× subgroup variable) into the full dataset and calculated interaction P-values. No correction for multiple comparisons was applied as all subgroup analyses were exploratory. The results are presented as forest plots.

Several sensitivity analyses were performed to verify the robustness of the observed associations. First, because the denominator of the reduction rate was the maximum diameter before treatment, we attempted to remove this variable from Model 3 and re-fit the regression model. In addition, PNI and GGT levels were adjusted based on Model 3. The PNI is a composite index that reflects the nutritional and immune status of patients, while GGT is related to oxidative stress and glutathione metabolism, both of which have been reported to be related to the prognosis of tumor radiotherapy and chemotherapy in previous literature (20–25). With the above analysis, it is possible to further verify whether the effect of LMR is independent of the nutritional status and oxidative stress pathways. For all statistical analyses, we used R and Free Statistics software (versions 4.2.2 and 2.5.1, respectively). The significance level was set at P < 0.05 (two-sided).

## 3 Results

### 3.1 Characteristics of the study population

In total, 247 patients were included in the final analysis. The baseline characteristics of the study population are summarized in **Table 1**. The median age was 63.7 years, and the majority of the patients had FIGO stage IIIA-IVA disease (67.6%). More than half of the patients (59.9%) received concurrent chemoradiotherapy. The median LMR after winsorization was 4.68 (IQR: 3.8-5.9).

**Table 1:** Baseline characteristics of the study population.

Variables	Total (n = 247)
Age, years, Mean ± SD	63.7 ± 9.8
BMI, kg/m², Mean ± SD	22.9 ± 3.5
Stage, n (%)	
FIGO IIA–IIB	80 (32.4)
FIGO IIIA–IVA	167 (67.6)
Lymph node metastasis, n (%)	
Negative	156 (63.2)
Positive	91 (36.8)
Concurrent chemoradiotherapy, n (%)	
No	99 (40.1)
Yes	148 (59.9)
Radiotherapy dose, n (%)	
45.0-47.5Gy	100 (40.5)
47.6-50.0Gy	147 (59.5)
Pretreatment max. diameter, cm, Median (IQR)	4.3 (3.5, 5.5)
LMR, Median (IQR)	4.68 (3.8, 5.9)
Tumor shrinkage rate, Median (IQR)	0.6 (0.4, 0.8)

The LMR was winsorized at the 1st and 99th percentiles to minimize the influence of extreme outliers. Data are presented as the mean ± SD, n (%), or median (IQR). LMR, lymphocyte-to-monocyte ratio; BMI, body mass index; IQR, interquartile range; SD, standard deviation.

### 3.2 Multivariable regression analyses

Multivariate linear regression demonstrated that LMR was independently and positively associated with the continuous tumor shrinkage rate (**Table 2**). In the Crude model without adjusting for any variables, each unit increase in LMR was associated with an average increase in tumor shrinkage rate of 0.018 (95%CI 0.001-0.034, P = 0.0413). After adjusting for age and BMI (Model 1), the β value was 0.018 (95%CI 0.001-0.035, P = 0.0415). After further adjustment for stage, maximum diameter before treatment, and lymph node metastasis (Model 2), the β value was 0.018 (95%CI 0.000-0.035, P = 0.0477). After adding concurrent chemoradiotherapy and radiotherapy doses (Model 3), the β value was 0.018 (95%CI 0.001-0.036, P = 0.0477). These results suggest that LMR is an independent protective factor for tumor shrinkage, and that this association is not affected by demographic factors, tumor burden, or treatment-related confounding factors.

**Table 2:** Multivariable linear regression analysis of the association between LMR and continuous tumor shrinkage rate.

Model	Adjustments	β (95% CI)	P-value
Crude	Unadjusted	0.018 (0.001–0.034)	0.0413
Model 1	Age, BMI	0.018(0.001–0.035)	0.0415
Model 2	Model 1 + FIGO Stage, Pre-treatment max. diameter, Lymph node metastasis	0.018 (0.000–0.035)	0.0477
Model 3	Model 2 + Concurrent chemoradiotherapy, Radiotherapy dose	0.018 (0.001–0.036)	0.0477

Data are presented as regression coefficients, β (95% confidence intervals). Model adjusted for age, BMI, FIGO stage, lymph node metastasis, concurrent chemoradiotherapy, radiotherapy dose, and pretreatment maximum diameter. LMR, lymphocyte−to−monocyte ratio; BMI, body mass index.

A significant response to radiotherapy was defined as a tumor reduction rate ≥70%. Multivariate logistic regression analysis showed that LMR was independently associated with significant response to RT (**Table 3**). In the crude model, every one-unit increase in LMR was associated with a 16.2% increase in the odds of a significant response (odds ratio (OR) = 1.162, 95% confidence interval [CI]: 1.017–1.327, P = 0.0271). In the fully adjusted model (adjusted for age, BMI, FIGO stage, maximum diameter before treatment, lymph node metastasis, concurrent chemoradiotherapy, and radiation dose), the odds of achieving a significant response increased by 11.7% (odds ratio [OR] =1.117, 95%CI 1.002-1.356, P = 0.0241) for each unit increase in LMR. These results further support the hypothesis that LMR are independently associated with a more complete radiotherapy response.

**Table 3:** Multivariable logistic regression analysis of the association between LMR and marked response (shrinkage rate ≥70%).

Model	Adjustments	Event, n/N (%)	OR (95% CI)	P-value
Crude	Unadjusted	78/247 (31.6)	1.162 (1.017–1.327)	0.0271
Model 4	Fully adjusted	78/247 (31.6)	1.117 (1.002–1.356)	0.0241

Data are presented as odds ratios (OR) with 95% confidence intervals (CI). Model adjusted for age, BMI, FIGO stage, lymph node metastasis, concurrent chemoradiotherapy, radiotherapy dose, and pretreatment maximum diameter. A marked response was defined as a tumor shrinkage rate of ≥70%. LMR, lymphocyte-to-monocyte ratio.

### 3.3 Linearity assessment

**Figure 2:**
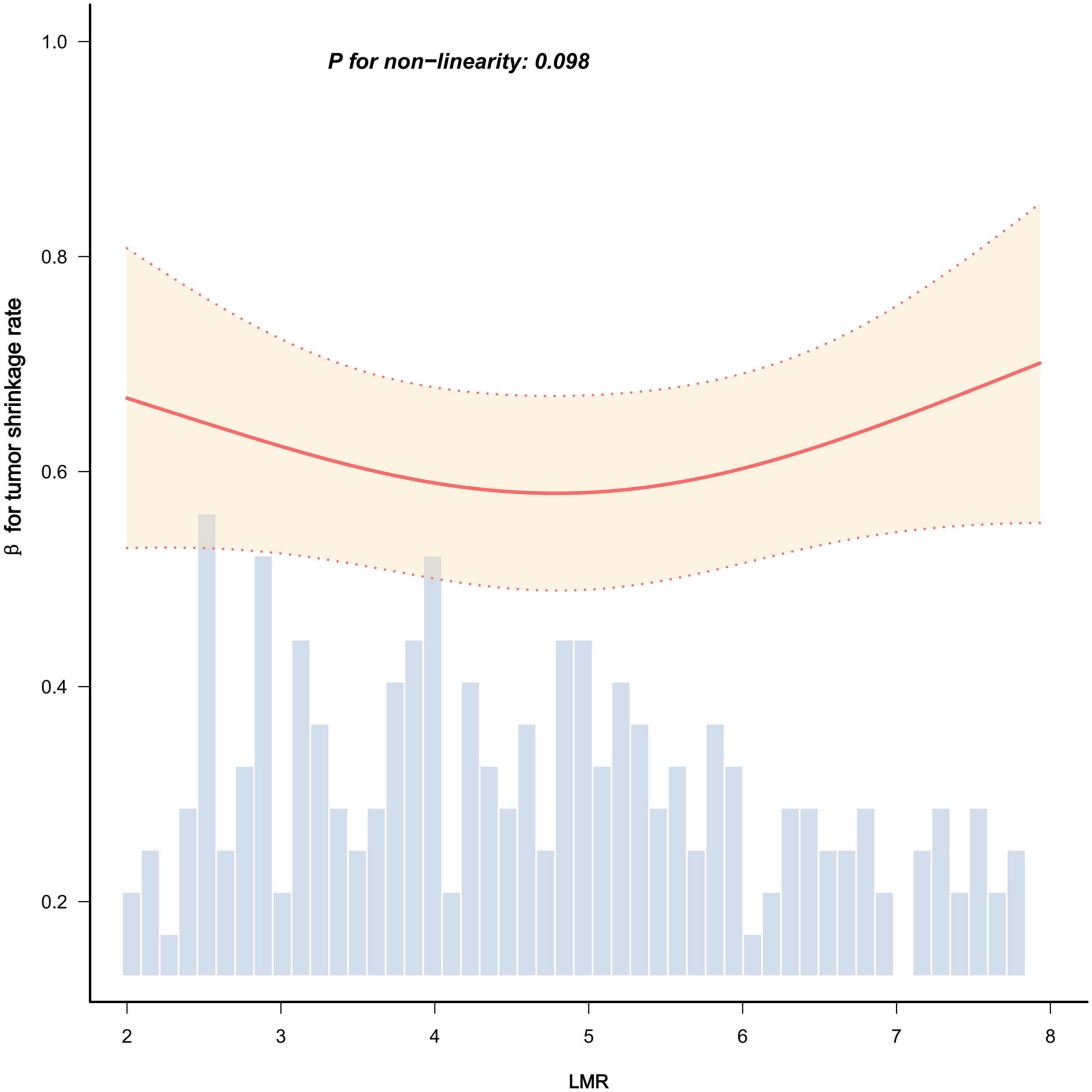
Restricted cubic spline curve illustrating the relationship between LMR and tumor shrinkage rate. The solid line represents the adjusted tumor shrinkage rate and the shaded area indicates the 95% confidence interval. The light-blue histogram shows the distribution of LMR among the study participants. The model was adjusted for age, body mass index (BMI), FIGO stage, lymph node metastasis, pretreatment maximum diameter, concurrent chemoradiotherapy, and radiation dose. The nonlinear term was not significant (P = 0.098), indicating a linear relationship.

We also verified the linear relationship with restricted cubic splines, with a nonlinear test of P = 0.098 (**Figure 2**), indicating a linear relationship between the LMR and tumor shrinkage rate.

### 3.4 Subgroup analysis

**Figure 3:**
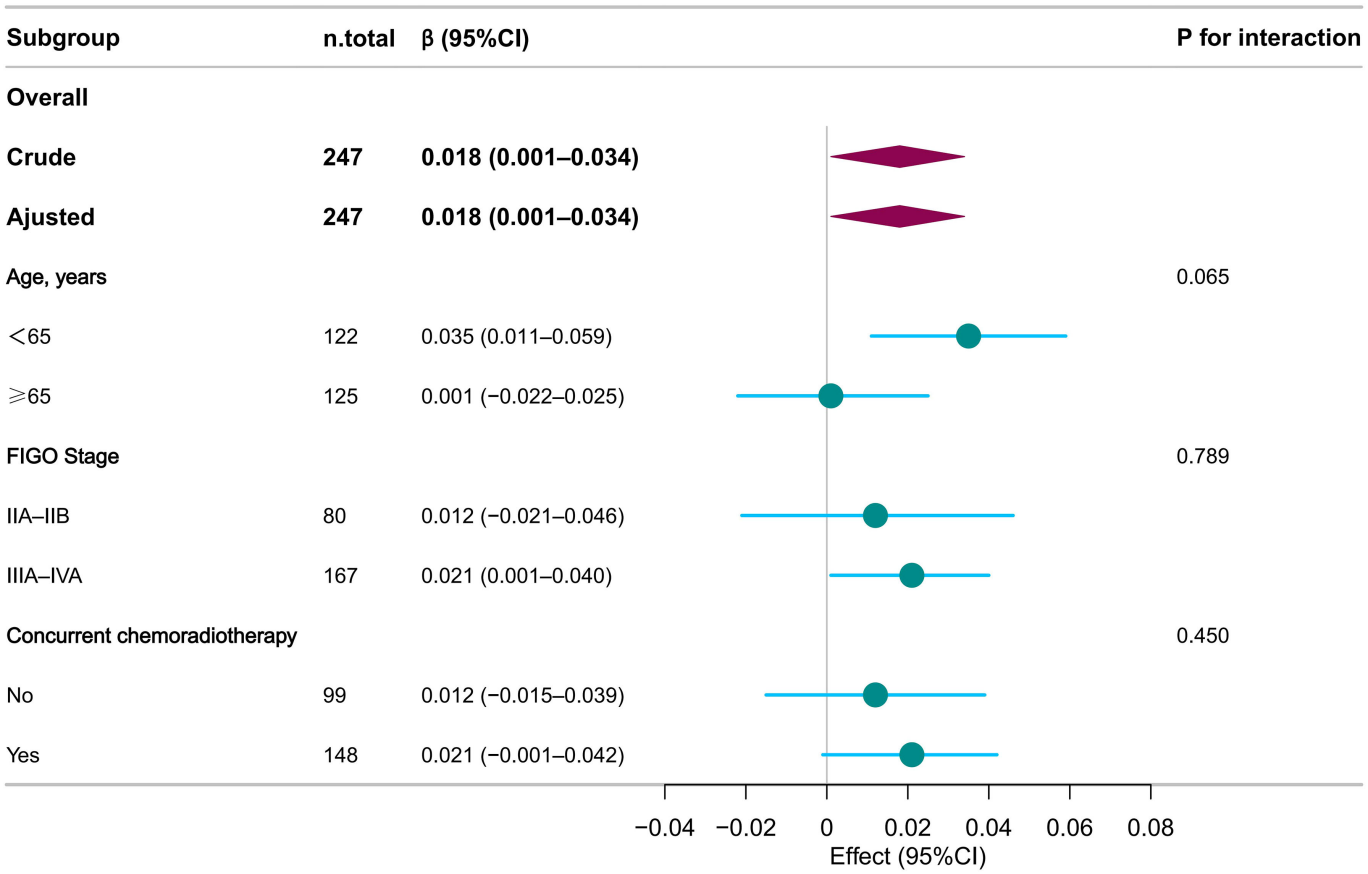
Forest plot of subgroup analysis for the association between LMR and tumor shrinkage rate. The β coefficients and 95% confidence intervals for each subgroup were derived using the same model as in Model 3. Interaction P−values obtained from likelihood ratio tests are shown on the right. CI, confidence interval.

The forest plot of the subgroup analysis (**Figure 3**) showed that the effect of the LMR was positive in all subgroups. The effect of age <65 years was significant (β=0.035, 95%CI 0.011-0.059), but that of age ≥65 years was not significant (β=0.001, 95%CI -0.022-0.025). However, the P value for interaction was close to, but did not reach statistical significance (P for interaction = 0.065), suggesting that age might have a certain effect modification effect, which needs further research to verify. In the subgroup of stage, the effect of LMR was significant in patients with advanced-stage disease (FIGO IIIA-IVA) (β=0.021, 95%CI 0.001-0.040), The effect of early stage disease was not significant, but P for interaction =0.789. In the concurrent chemotherapy subgroup, the direction of the LMR effect was consistent regardless of whether the patients received concurrent chemotherapy, and the P for interaction was 0.450. Overall, the effect of the LMR remained stable across subgroups.

### 3.5 Sensitivity analysis

**Supplementary Table 1** shows the results of the sensitivity analysis. Excluding the maximum diameter before treatment based on Model 3, the effect of the LMR was slightly enhanced (β=0.019, 95%CI 0.002-0.036, P = 0.0301). After additional adjustment for PNI, the LMR effect remained significant (β=0.020, 95%CI 0.001-0.038, P = 0.0350). After additional adjustment for GGT, the LMR effect did not change significantly (β=0.018, 95%CI 0.000-0.035, P = 0.0479). These results support the robustness of the main conclusions of this analysis.

When the tumor shrinkage rate was log-transformed and re-analyzed using the same covariate adjustment as in Model 3, the association showed a consistent positive direction (β = 0.183, 95% CI: -0.008–0.373, P = 0.0616).

In addition, ROC analysis showed an AUC of 0.579 (95% CI: 0.488–0.670). The Youden index indicated a cutoff of 0.85 for the predicted probability, with a sensitivity of 41.5% and specificity of 77.5%. These figures suggest that, on its own, LMR is not a strong classifier, which is in line with our view that it should be considered a predictor associated with response, rather than a test that can classify patients by itself. The ROC curve is shown in **Supplementary Figure 2**. Taken together, these findings support LMR as an independent predictor of tumor shrinkage, although its modest discriminative ability suggests that it should be used alongside other clinical parameters rather than in isolation.

## 4 Discussion

### 4.1 Principal findings

The present study identified pretreatment LMR as an independent predictor of superior tumor reduction after external beam radiotherapy in 247 patients with locally advanced cervical cancer. For each unit increase in the LMR, the tumor shrinkage rate increased by 1.8 percentage points (β=0.018, 95%CI 0.001-0.036). This association remained stable after adjusting for age, BMI, stage, maximum diameter before treatment, lymph node metastasis, concurrent chemoradiotherapy, and radiation dose. A higher LMR was also associated with a higher likelihood of significant radiation response (≥70% reduction). Subgroup analysis showed that the LMR effect was consistent in each clinical subgroup, and the sensitivity analysis supported the robustness of the conclusion. These results suggest that pretreatment LMR levels, as a reflection of systemic inflammatory status, may serve as a simple and readily available reference index for radiosensitivity.

### 4.2 Biological mechanisms

The relationship between LMR and radiotherapy response has a clear biological basis, mainly involving bidirectional regulation of the immune system by radiotherapy. Radiotherapy can cause direct damage to lymphocytes, particularly after large-field pelvic irradiation, and the number of circulating lymphocytes is significantly reduced. An increased risk of worse overall survival was confirmed to be associated with radiation-induced lymphopenia (RIL) in a meta-analysis of 56 studies (13,223 patients; HR = 1.70, 95% CI: 1.55–1.86, P < 0.01) (26). A cervical cancer-specific meta-analysis comprising 952 patients further confirmed that RIL independently correlated with worse overall survival (HR = 2.67) and progression-free survival (HR = 2.17) (27). Patients with low LMR before treatment have limited lymphocyte reserves. After large-field pelvic external beam irradiation, further lymphocyte depletion may be detrimental to the radiation-induced immune response, thereby affecting tumor shrinkage.

The role of monocytes in radiotherapy is complex. Tumor-associated macrophages are considered immunosuppressive cells. However, recent studies have found that in the clinical conformal radiotherapy mode, radiotherapy can rapidly recruit monocytes into the tumor microenvironment, and these monocytes upregulate MHC-II and costimulatory molecules to activate CD8+ T cells, thereby playing an anti-tumor role (28). However, monocytes may also differentiate into immunosuppressive cells (such as CD301b+ monocyte-derived dendritic cells) that mediate radiation resistance (29). The observation that a low LMR (relatively few lymphocytes and relatively more monocytes) is associated with poor tumor shrinkage response supports the possibility of a dominant tumor-promoting role of monocytes in cervical cancer radiotherapy at the macro level.

There may be a synergistic effect between the DNA damage response induced by radiotherapy and the systemic immune status. It has been reviewed that DNA double-strand breaks are the core mediator linking radiotherapy with anti-tumor immune activation (30). by inducing immunogenic cell death, releasing tumor-associated antigens, and remodeling the tumor microenvironment, radiotherapy elicits systemic anti-tumor immune responses (31). Patients with a higher LMR may have a more favorable systemic immune profile; however, this hypothesis requires further mechanistic investigation. We do not claim that the LMR itself plays a specific causal role; rather, we interpret it as a reflection of the patient’s systemic inflammatory and immune status.

### 4.3 Comparison with previous studies

As an inflammatory immune indicator, LMR have been confirmed to have prognostic value in radiotherapy/chemoradiotherapy for various tumors. In a meta-analysis of 11 studies including 3,377 esophageal cancer patients, low pretreatment LMR was significantly associated with worse overall survival (HR = 1.65) and progression-free survival (HR = 1.58) (32). In laryngeal cancer, patients who exhibited an increase in LMR after two weeks of radiotherapy had a significantly higher 5-year overall survival rate than those without a notable increase (57% vs. 31%, P = 0.015) (33), suggesting that dynamic changes in LMR may have more prognostic value than a single measurement, which also echoes the limitations of this study. Radiotherapy-induced low LMR is a robust prognostic marker of recurrence and survival outcomes in breast cancer (34). A pooled analysis of 17 studies involving nearly 8,000 patients with bladder cancer revealed that low LMR was significantly associated with worse overall survival (HR = 1.56), recurrence-free survival (HR = 1.74), and progression-free survival (HR = 2.04) (35). Furthermore, in head and neck squamous cell carcinoma, low LMR, considered both as a continuous variable and dichotomized variable, was consistently associated with worse overall survival (OS) and cancer-specific survival (CSS) (36). These previous studies are largely consistent with the current findings and jointly reinforce the concept that a high LMR, indicative of a favorable anti-tumor immune profile, is associated with a more favorable response to radiation therapy/chemoradiotherapy.

Prior investigations in the field of cervical cancer have primarily explored the link between LMR and long-term outcomes. A 2020 study found that a higher pretreatment LMR was a positive prognostic indicator in patients receiving radical chemoradiotherapy for cervical cancer (37). Another retrospective study conducted in 2024 confirmed that both PNI and LMR are reliable prognostic indicators for cervical cancer, and that monitoring these markers contributes to more precise risk stratification (17). However, few studies have focused directly on the early tumor shrinkage rate after external beam irradiation. The innovation of this study is that it advances the endpoint of short-term tumor regression after external-beam irradiation rather than waiting for a survival outcome of months or years, which provides a more timely basis for the early identification of radiotherapy-insensitive patients.

### 4.4 Clinical significance

LMR are derived from routine blood tests, which add almost no additional costs and are easy to promote in hospitals at all levels. According to the findings of this study, patients with a low LMR have relatively poor tumor shrinkage after external beam radiotherapy, suggesting that these patients may belong to a high-risk group with a poor response to radiotherapy. It is worth exploring intensive treatment strategies in future prospective studies. Our team previously demonstrated that nimotuzumab combined with chemoradiotherapy can improve the tumor reduction rate of EBRT in elderly patients with locally advanced cervical cancer (8), suggesting that more aggressive treatment strategies for people with low LMR may have certain clinical value. In addition, the LMR can be used as a component of multidimensional prediction models. Combined with the ongoing radiomics research conducted by our group, the findings of this study are expected to inform the development of improved predictive tools for the response to radiotherapy.

Admittedly, the AUC of 0.579 was modest, but this was hardly surprising for a single blood parameter. Radiotherapy response is determined by a mix of tumor biology, host immunity, and treatment factors; therefore, we would not expect LMR to be a perfect discriminator on its own. What makes LMR useful is not its standalone diagnostic power but its simplicity, low cost, and the fact that it can be easily combined with other clinical or imaging information in future studies.

### 4.5 Study strengths and limitations

This study has several methodological strengths. Notably, the relatively large sample size (247 patients) yielded sufficient statistical power for a single-center retrospective analysis, with *post-hoc* power analysis demonstrating >80% power. Second, we adjusted covariates sufficiently to include not only the usual age, BMI, stage, nodal metastasis, concurrent chemoradiotherapy, radiation dose, and maximum diameter before treatment to minimize the effect of tumor burden on shrinkage. In addition, both the continuous outcome (tumor shrinkage rate) and binary outcome (tumor shrinkage rate ≥70%) were analyzed, and the results were consistent and mutually confirmed. The effect of LMR after adjusting for PNI and GGT levels in the sensitivity analysis enhanced our confidence in our conclusions.

Our study had some limitations. The results of this single-center retrospective study should be interpreted with caution because of inevitable selection bias. Further prospective multicenter studies are required to assess the generalizability of these conclusions. In addition, we measured the LMR only once before treatment and did not track dynamic changes during radiotherapy. Studies on laryngeal cancer suggest that an elevated LMR during radiotherapy may predict a better prognosis. Thus, dynamic monitoring can provide additional information. In our analysis, adjustment for HPV status was not made because HPV testing was not performed uniformly in the early days of data collection and most patients did not have complete HPV data. This is a limitation of the present study. HPV status, especially HPV 16/18, compared to other high-risk types, may affect the radiosensitivity of cervical cancer and may play a role as a residual confounding factor. If HPV positivity was more common in the high LMR group, the association observed may have been overestimated. In contrast, if HPV status is balanced across the LMR groups, the confounding effect is small. Given that the association between LMR and tumor shrinkage remained significant after adjusting for multiple established prognostic factors, including stage, tumor size, nodal status, and treatment-related variables, we believe that the independent effect of LMR is unlikely to be entirely driven by HPV. Nonetheless, prospective studies with uniform HPV testing are required to confirm our findings. The P value for the interaction by age subgroup was close to 0.05 (P = 0.065), suggesting that age may modify the effect to some extent, but this needs to be further confirmed by a larger sample. In addition, tumor shrinkage was assessed using the RECIST 1.1 maximal-diameter approach rather than volumetric measurement. The maximum tumor diameter method is a standard clinical method and has practical advantages in routine clinical practice, whereas volume assessment can more comprehensively capture tumor regression, especially for shrinking tumors with irregular morphology. This methodological difference should be considered when interpreting the results. Finally, it is difficult to rule out unknown or unmeasured confounding variables in retrospective studies. Therefore, these findings should be cautiously interpreted.

## 5 Conclusion

In conclusion, the pretreatment LMR level is independently associated with the tumor shrinkage rate after external beam radiotherapy in patients with locally advanced cervical cancer, and a higher LMR is associated with a better tumor regression response. This finding suggests that LMR may be associated with radiosensitivity, but prospective, multicenter studies are needed to verify this finding. Future studies should explore the combined predictive value of LMR, radiomics, and molecular markers.

## Data Availability

The data that support the findings of this study were obtained from the electronic medical record system of Zhangzhou Affiliated Hospital of Fujian Medical University. Due to patient privacy and confidentiality regulations, the raw data are not publicly available. De−identified data may be made available from the corresponding author upon reasonable request and with institutional approval. Requests to access these datasets should be directed to Yanhong Zhuo, zhuoyanhongfj@163.com.
