# Supplementary material for: Higher pre-treatment lymphocyte-to-monocyte ratio predicts better tumor shrinkage after external beam radiotherapy in locally advanced cervical cancer
Source: Front Oncol. 2026 Jul 22;16:1917302. doi: 10.3389/fonc.2026.1917302 (PMC13437358; doi:10.3389/fonc.2026.1917302)
Supplement: Supplementary file 1 [file DataSheet1.docx]

Supplementary Material

# 1 Supplementary Figures and Tables

## 1.1 Supplementary Figure

**Supplementary Figure S1. Boxplot of LMR values.**

LMR was winsorized at the 1st and 99th percentiles to reduce the influence of extreme outliers. The box represents the interquartile range (IQR) from Q1 (3.26) to Q3 (6.02), with the horizontal line inside indicating the median (4.68). The whiskers extend to the minimum and maximum values. No outliers were detected. LMR, lymphocyte‑to‑monocyte ratio.

**Supplementary Figure S2. Receiver operating characteristic (ROC) curve for LMR in predicting marked tumor shrinkage (≥70%).**

The area under the curve (AUC) was 0.579 (95% CI: 0.488–0.670). The optimal cut-off value determined by the Youden index was a predicted probability of 0.85, with a sensitivity of 41.5% and specificity of 77.5%.

## 1.2 Supplementary Table

# **Supplementary Table S1. Sensitivity analyses of the association between LMR and tumor shrinkage rate.**

| **Model** | **Adjustments** | **β (95% CI)** | **P-value** |
| --- | --- | --- | --- |
| **Model 3** | Fully adjusted | 0.018 (0.001–0.036) | 0.0477 |
| **Model 5** | Model 3 without the maximum diameter before treatment | 0.019 (0.002–0.036) | 0.0301 |
| **Model 6** | Model 3 + PNI | 0.020 (0.001–0.038) | 0.0350 |
| **Model 7** | Model 3 + GGT | 0.018 (0.000–0.035) | 0.0479 |
| **Model 8** | Logit-transformed TSR | 0.183 (-0.008–0.373) | 0.0616 |

**Note:** Model 3 were adjusted for age, BMI, FIGO stage, lymph node metastasis, pre-treatment maximum diameter, concurrent chemoradiotherapy, and radiotherapy dose (Model 3).

**Abbreviations:** BMI, body mass index; GGT, gamma-glutamyl transferase; PNI, prognostic nutritional index; TSR denotes tumor shrinkage rate.
